# Influence of Salting Method on the Chemical and Texture Characteristics of Ovine Halloumi Cheese

**DOI:** 10.3390/foods8070232

**Published:** 2019-06-29

**Authors:** Stelios Kaminarides, Ekaterini Moschopoulou, Fotini Karali

**Affiliations:** Laboratory of Dairy Research, Department of Food Science and Human Nutrition, Agricultural University of Athens, Iera Odos 75, 118 55 Athens, Greece

**Keywords:** Halloumi cheese, cheese salting, cheese texture, cheese inorganic fraction

## Abstract

The effects of dry salting for 24 h, or brine salting under different conditions (i.e., 7%, 10%, or 13% NaCl (*w*/*w*) at 4 or 20 °C for 3, 6, 24, or 48 h) on ovine Halloumi cheese composition and textural properties were studied. In the brine-salted cheeses, the moisture content, ranging from 45.53 ± 0.7 to 53.55 ± 0.6 g/100 g, was decreased as the concentration and temperature of brine and salting time were increased. In contrast, the salt content, ranging from 2.17 ± 0.01 to 4.97 ± 0.10 g/100 g, increased by increasing the salting time and brine concentration, and the increased sodium content of cheeses was correlated with their decreased potassium content. Cheeses dry-salted for 24 h contained higher levels of calcium (1064–1093 mg/100 g) than brine-salted cheeses kept for 24 or 48 h (751–922 mg/100 g). The same trend was observed for phosphorus, magnesium, and potassium content. In addition, dry-salted cheeses showed significantly (*p* < 0.05) lower hardness and fracturability values, compared to cheeses brine salted at 13% brine for more than 24 h, independently of the brine temperature. It was concluded that dry salting of Halloumi cheese for one day was the most appropriate salting method for dietary and nutritious reasons.

## 1. Introduction

Halloumi, a very popular cheese in Eastern Mediterranean countries, has been traditionally produced in Cyprus for hundreds of years. The Committee for Standards of the Cyprus Ministry of Commerce and Industry [1] recognizes two types of Halloumi cheese, ‘fresh’ and ‘mature’ Halloumi cheese, for which the definitions and standards have been established. Fresh Halloumi cheese is sold immediately after production and has a mild, milky flavor; an elastic and semi-solid texture without holes; and can be easily sliced. Mature Halloumi cheese has a salty and moderately acidic taste, a firm texture, and a tough body. A characteristic of Halloumi cheese manufacture is that the blocks of curd are heated at 90–95 °C in deproteinized whey for at least 30 min. Halloumi cheese is a semi-hard rindless cheese and is traditionally made from a mixture of ovine and caprine milk, although nowadays large dairies use almost exclusively bovine milk. In the traditional process, the curd is initially not pressed at all, and the fresh cheese is dry salted and sprinkled with dry, crushed leaves of mint (*Mentha viridis*) and then kept in salted whey (12% NaCl) at low temperature for a prolonged period of time until consumed. In the industrial process, however, the curd is pressed at a pressure of 0.35–0.40 MPa and then cut into blocks (10 cm × 10 cm × 3 cm) which are transferred to hot whey and cooked at 90–92 °C for 30 min. The way in which the cheese is salted also differs in industrial production. The cooked cheese blocks are not dry salted, but left to cool in whey brine (12% NaCl) at 4 °C for ~18 h and then sprinkled with dry, sterilized mint; packed under vacuum in polyethylene bags; and then stored in refrigerated stores at 4 °C until retailed. Mature Halloumi in whey brine may be stored for at least 40 days at 15–20 °C [2], and a significant proportion of the total Halloumi cheese production is preserved in this way. However, storage in brine changes cheese properties such as the organic acids profile, volatile aroma compounds, and sensory characteristics [3]. 

The salt content of cheese varies according to the method of salting, the cheese type, cheese geometry, cheese size, etc., and this affects not only its flavor but also most of its physicochemical characteristics [4,5]. Although Halloumi cheese can be salted in different ways, the literature concerning the effects of salting on its characteristics is limited to the substitution of sodium chloride by potassium chloride [6,7,8,9,10]. The aim of this study is to evaluate the effects of various methods of salting (dry or brine salting under various sodium chloride concentrations, brining temperatures, and brining times) on the chemical and textural characteristics of ovine Halloumi cheese, and to recommend the most suitable salting method for its manufacture. 

## 2. Materials and Methods 

### 2.1. Cheesemaking

**Figure 1:**
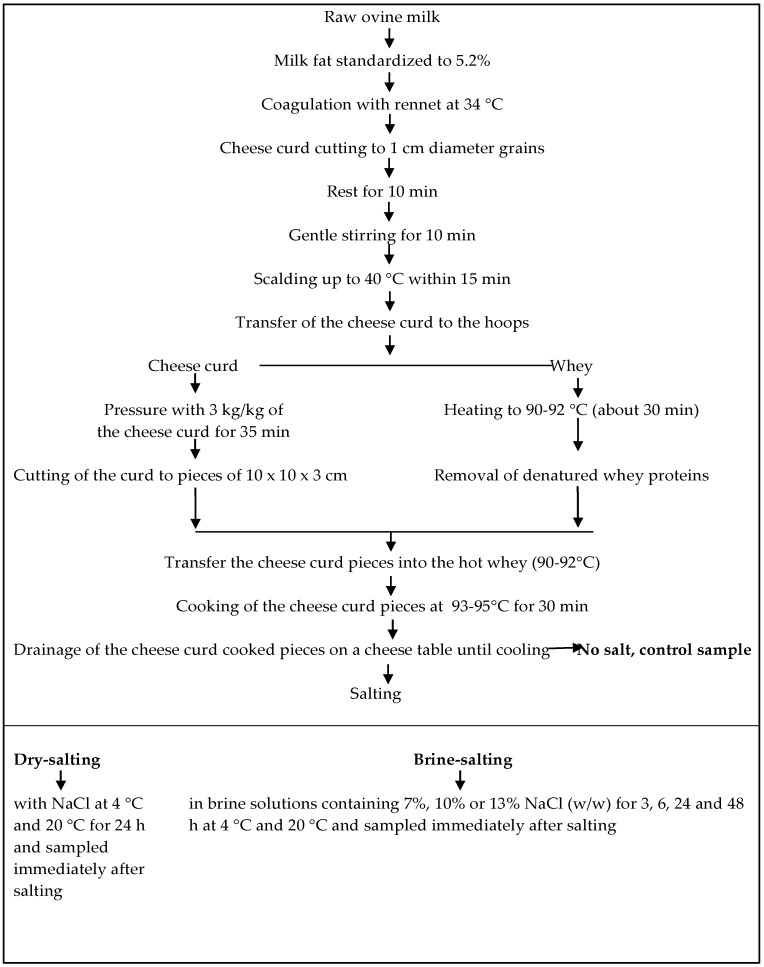
Flow diagram of ovine Halloumi cheese production, including the stages of sampling.

Halloumi cheese was manufactured in a cheese pilot plant according to the cheesemaking procedure described by Anifantakis and Kaminarides [11], using local ovine milk. The flow diagram of the cheesemaking is shown in Figure 1. After cooking, the cheese curd blocks, sized 10 × 10 × 3 cm, were salted in two different ways: a) By dry salting with coarse NaCl (2% of curd weight) onto all sides of the cheese block for 24 h without the addition of dried *Mentha viridis* so as not to affect analyses, or b) by brine salting. In the latter case, cheese blocks were immersed into brine solution containing 7%, 10%, or 13% NaCl (w/w) and kept at 4 and 20 °C for 3, 6, 24, and 48 h. In all cases, the ratio of brine volume to cheese weight was 2.5:1. Control cheese was prepared without salting and stored at 4 °C. Three cheesemaking trials were conducted, and cheese samples were subjected to the following analyses. 

### 2.2. Chemical Analyses

Before analyses, the brine-salted cheeses were removed from the brine and drained for 2 min. All cheeses were analyzed for the total solids content by heating at 102 °C [12], and for the ash content as specified in the AOAC method [13]. Acidity was determined by a titration method using 0.11 N NaOH solution. The NaCl content was determined by the potentiometric titration method [14]. Calcium, magnesium, potassium, and sodium contents were determined by the Atomic Absorption Spectrometry method [15] on a Shimadzu AA-6800 Atomic Absorption Spectrophotometer (Shimadzu AA-6800, Kyoto, Japan) equipped with the autosampler Shimadzu ASC-6100 and the software WizAArd v. 2.30. Phosphorus content was determined by the molecular absorption spectrometry [16]. All analyses were performed in duplicate. 

### 2.3. Assessment of Textural Properties

The textural properties of the cheeses were measured by a Shimadzu testing instrument, model AGS-500 NG (Shimadzu Corporation, Kyoto, Japan), equipped with a 5 kg load cell. A plunger with a diameter of 6 mm was attached to the moving crosshead, and analysis speed was set at 2.5 cm min-1 in both up and down directions. Data were obtained for Hardness (N), Fracturability (N.mm), Adhesiveness (N.mm), Elasticity (mm), and Cohesiveness (N.mm) as described by Kaminarides and Stachtiaris [17]. 

### 2.4. Statistical Analysis

The obtained data were subjected to analysis of variance (ANOVA) using the software Statgraphics (Statistical Graphics Corp. Rockville, Maryland, MD, USA). A split–split plot design was used, and paired comparisons of means were made using the Duncan test (*p* < 0.05). 

## 3. Results and Discussion

### 3.1. Chemical Composition 

The chemical compositions of experimental Halloumi cheeses salted by various methods are presented in Table 1. It is obvious that salting in brine with different NaCl concentrations and temperatures significantly (*p* < 0.05) affected the moisture contents of the cheeses. In the case of dry salting, the salted cheeses kept at 4 and 20 °C presented significantly (*p* < 0.05) less moisture content compared to unsalted cheeses stored at 4 °C. Among the cheeses that were salted in brine, those kept at 20 °C showed significantly (*p* < 0.05) lower moisture contents than the cheeses kept at 4 °C. 

According to Geurts et al. [18], during salting in brine, the water bounded on cheese decreases with temperature increase. The experimental cheeses immersed into 7% brine, especially those stored at 4 °C, showed a slight continuous increase of moisture content during the salting period. The moisture content of the cheeses maintained in 10% brine at 4 and at 20 °C was not significantly (*p* > 0.05) affected by salting time. In contrast, it was significantly (*p* < 0.05) influenced by the brine concentration. A progressive decrease in the moisture content was observed as brine concentration increased. At a low brine concentration (e.g., 7% NaCl), water is transferred from the brine into the cheese to achieve osmotic pressure equilibrium. In contrast, the cheese maintained in 13% brine loses water. In general, the decreases of moisture contents of all cheeses (Table 1) were due to the absorption of NaCl, and, in parallel, due to the removal of water from their mass. It is known that cheese moisture loss and the corresponding cheese weight reduction are some of the negative consequences of brining, and the denser the brine, the higher the cheeses’ weight loss. Consequently, salting decreases or increases the Halloumi cheese moisture content according to the quantity of added salt. Similar results have been reported for other semi-hard or hard cheeses during brining [19,20]. 

Furthermore, significant (*p* < 0.05) differences in the salt contents of cheese samples were observed among the different methods of salting and salting times (Table 1). Salt contents of surface dry-salted cheeses stored at 4 or 20 °C were significantly (*p* < 0.05) higher than those of unsalted cheeses. In the case of brine salting, cheeses maintained in brines of 7%, 10%, or 13% NaCl at 4 or 20 °C showed rather proportional increased salt contents. During brining, the quantity of NaCl that is absorbed by the cheese, and consequently the cheese sodium content, depends on several parameters such as brine concentration, salting time, cheese moisture, and cheese pH. In general, NaCl moves from brine into the cheese, and, in parallel, water diffuses out of the cheese matrix to achieve the osmotic pressure equilibration [5]. The increases of salt contents in the experimental cheeses were faster during the first hours of salting, and slower after 24 h of maintenance. This was explained by the fact that the rate of salt absorption decreases with time, because the difference in salt content between the brine and the cheese moisture decreases [4,21,22]. Finally, since salt is the main component of ash, similar significant (*p* < 0.05) differences in the ash contents of cheeses were observed among the various conditions of salting. 

Statistically significant (*p* < 0.05) differences in acidities of Halloumi cheese samples were observed between the brine temperatures and brine salting times. The acidity was higher in cheeses salted at 20 °C than in the cheeses salted at 4 °C. In addition, the acidity was higher in dry-salted cheeses than in brine-salted cheeses, decreasing with the brining time. This was attributed to the progressive increase of moisture contents of the cheeses maintained in brine, with a parallel diffusion of lactic acid in brine. Kamleh et al. [10] reported a significant effect of brining time with brine solution of 10% NaCl on the lactic acid content of Halloumi cheese. In general, acidity was attributed to microbial growth, especially of the survived non-starter lactic acid bacteria (NSLAB). Despite the high temperature of cooking, i.e., at 92 °C for 30 min, Halloumi cheese contains several groups of microorganisms, and it has been shown that NSLAB in Halloumi cheeses stored in different brine solutions increase significantly, independently of the brine solution composition [10].

### 3.2. Inorganic Elements 

The concentrations of the inorganic elements in Halloumi cheese in relation to the salting treatments are shown in Table 2. As expected, sodium content was significantly (*p* < 0.05) affected by the duration of brining. The most significant increase in sodium content was observed between the first hours of brining. Moreover, sodium contents in the examined Halloumi cheeses increased as brine concentration increased. Regarding the effect of temperature, sodium content was linear (y = 155.24x + 861.7, *R^2^* = 0.978) and increased constantly at 20 °C as a function of salting time. In contrast, during brining with 7% NaCl at 4 °C, the linearity was lower (y = 125.18x + 954.63, R^2^ = 0.862) due to the fluctuation in Na content with time and the rapid increase in sodium content between 6 and 24 h. However, in 13% brine, the sodium content of cheese in relation to time had a continual increase, but was non-linear because the entry of salt into the cheese took place at a reduced rate [19]. Additionally, the initial (3 h) Na concentration was much higher at 13% NaCl, irrespective of temperature. Brining at low temperatures decreases the rate of sodium penetration into cheese, because protein hydration is favored, and consequently the free water available in the cheese moisture for the movement of sodium decreases [23,24]. Finally, the sodium contents of cheeses, determined by the atomic absorption spectrometry method, were highly correlated (y = 0.894x + 0.042, *R^2^* = 0.935) to the NaCl contents of cheeses determined by the potentiometric method. 

Regarding the potassium content, dry-salted cheeses contained significantly (*p* < 0.05) more potassium than brine-salted cheeses. In addition, potassium content was negatively affected by the salting time, as it was significantly decreased by up to 24 h of brining (Table 2). This was due to the loss of soluble potassium salts in brine, since 90%–95% of the potassium in milk and cheese is found in a soluble form as free ions. Compared to other mineral contents, potassium content varies in a different way, probably due to its high solubility and to the fact that Na^+^ and K^+^ ions can substitute each other. Therefore, the increase of sodium content in cheese causes a decrease of the potassium content. In fact, sodium and potassium contents were inversely proportional to the concentration of brining. Both the salting with brine of 10% concentration and the duration of brining have significant effects on the sodium and potassium contents of Halloumi cheese [10].

As far as calcium is concerned, 80% of this inorganic element found in the colloidal phase is associated to casein micelles, and the rest is associated to phosphoserines. The role of colloidal calcium phosphate (CCP) is very important, as CCP connects the submicelles to form casein micelles and contributes to the structure of the cheese protein matrix. The results showed that both the temperature of brine (4 or 20 °C) and the duration of brining in 7% NaCl (between 6 and 24 h) affected the calcium contents of the experimental cheeses (Table 2). Cheeses brined at 4 °C contained less calcium than cheeses brined at 20 °C. This result was attributed to the fact that, because at low temperatures colloidal calcium becomes soluble [25], the soluble calcium in cheese serum increased and finally diffused into the brine. In addition, colloidal calcium in cheese becomes soluble at pH < 5.6 and especially at pH of about 5.0 [26,27]. Diffusion of calcium in brine is a very common phenomenon in cheeses in brine, especially in Feta and white cheeses [28]. Moreover, an exchange between ions of calcium in the casein matrix of a cheese kept in brine and ions of sodium in brine has been clearly shown [5,20,24]. However, in this study, the pH of Halloumi cheeses was not an important factor of calcium solubility, since the pH of cheeses ranged within 6.15–6.38. The highest concentration of calcium was observed in the dry-salted cheeses, probably due to their lower moisture contents and hence their higher dry matter contents, and to a lack of any loss. In contrast, the brine-salted cheeses exhibited calcium content levels like the unsalted cheeses. 

Depending on the process technology and especially on the pH of cheese curd during draining, more than 50% of phosphorus content in cheese is attached to casein [29]. In the examined cheeses, the phosphorus content was significantly (*p* < 0.05) affected by the temperature of brine, by the interaction of brine concentration and temperature, and by the duration of brining (Table 2). Temperatures as low as 4 °C negatively influenced the phosphorus content, and the lowest value was observed during brining from 3 to 6 h. There were also significant differences between dry salting and brining, independently of the concentration of brine. As shown in Table 2, surface dry-salted cheeses contained more phosphorus than brine-salted cheeses. As in the case of calcium, this was due to partial diffusion of phosphorus into the brine. In general, the kinetic profiles of phosphorus content were similar to those of calcium, because the factors that affect the distribution of colloidal or soluble calcium and phosphorus in cheese are the same, although they do not act in the same way. Calcium from the colloidal phase moves to serum easier than phosphorus. In addition, soluble calcium is removed with a higher rate than soluble phosphorus during whey removal, because of the higher movement of calcium ions. This is due to the lower molecular weight of calcium ions compared to molecular weights of phosphates [30]. 

Magnesium is found in the cheese serum, and its concentration depends on the cheese technology. The magnesium contents of the examined Halloumi cheeses were significantly higher in dry-salted cheeses than in brine-salted cheeses (Table 2), and were also negatively affected by the duration of brining. This was because during dry salting the soluble magnesium salts remained in the cheese, while during brining, they diffused into brine. According to Gaucheron et al. [31], the absorption of NaCl at pH 6.0–6.65 has similar effects on the soluble magnesium as on the calcium. It seems that the mean pH value of 6.2 of the examined Halloumi cheeses enhanced the effect of NaCl on the ionic strength of cheese and caused significant solubility of magnesium. Therefore, part of the magnesium found in the colloidal phase became soluble like calcium and diffused into brine. 

### 3.3. Texture Profile

The results from texture analyses (Table 3) showed that hardness, fracturability, and adhesiveness of the cheeses were significantly (*p* < 0.05) affected by the different methods of salting. Dry-salted cheeses exhibited significantly (*p* < 0.05) lower hardness and fracturability values compared to brine-salted cheeses salted at 13% brine for more than 24 h, independently of the brine temperature. All cheeses kept at 20 °C presented higher values for hardness and fracturability properties than cheeses kept at 4 °C, probably due to their higher acidity values and total solids. It has been reported that high acidity and total solids content make cheeses harder and less easily deformed [32]. In addition, cheeses salted in brines of 7%, 10%, and 13% exhibited a continuous gradual increase in hardness and fracturability throughout storage in brine. Papademas [2] has also reported increases of fracturability during storage in brine, due to salt diffusion from brine to cheese and to water loss from cheese to brine. As mentioned before, this diffusion depends on the concentration of brine and the duration of brining. The loss of water from the cheese surface is higher in the case of brining with a high concentration of NaCl than in brining with a low concentration of NaCl. In addition, the penetration of salt into cheese is difficult in cheeses with dry surfaces and decreased porosity [5]. 

The dry-salted cheeses stored at 20 °C exhibited significantly (*p* < 0.05) lower values for adhesiveness than the cheeses stored at 4 °C. Regarding the adhesiveness of brine-salted cheeses, cheeses stored in 13% brine showed significantly (*p* < 0.05) higher values than cheeses stored in 7% brine. In contrast, dry-salted cheeses and brine-salted cheeses did not statistically differ in cohesiveness at both salting temperatures, even compared with the unsalted cheese controls. The same was true for the elasticity, although the obtained values were lower than the values reported for ovine Halloumi cheese [33]. Ayyash et al. [9] reported increased adhesiveness and decreased cohesiveness for Halloumi cheese kept in 18% brine at 4 °C for 56 days, due to decreased calcium content and increased proteolysis. 

As for elasticity, like calcium content, it was not affected by the brine concentration. According to Prentice et al. [34], the rheological role of casein in cheese is to provide a continuous elastic framework for the individual cheese granules. 

Regarding unsalted cheeses, values for hardness, cohesiveness, and elasticity were in agreement with other values reported for fresh unsalted Halloumi cheeses cooked for 30 min [33]. 

## 4. Conclusions

From the obtained results, it was concluded that the method of salting influenced the mineral content of ovine Halloumi cheese more, and the textural properties less. Potassium, calcium, magnesium, and phosphorus contents were significantly higher in dry-salted cheeses than in brine-salted cheeses, whereas sodium was lower. For these reasons, traditionally produced, dry-salted Halloumi cheese could be considered of greater dietary value than industrially manufactured, brine-salted Halloumi cheese. In addition, cheeses dry salted for 24 h exhibited significantly lower hardness and fracturability values compared to cheeses brine salted at 13% brine for more than 24 h, independently of the brine temperature. Regarding the brine-salted cheeses, cheese hardness and fracturability were more enhanced when kept at 20 °C than at 4 °C, independently of the brine concentration. 

## Figures and Tables

**Table 1:** Physicochemical characteristics of Halloumi cheese salted by different methods at 4 and 20 °C for different times (means of three trials ± SD).

Chemical Characteristics	No Salt	Salting Method	Temperature of Cheese Salting (°C)								Pairs of Means That Significantly Differed at (*p* ≤ 0.05)
			4				20				
			Salting Time (h)								
			3	6	24	48	3	6	24	48	
Moisture (%)	47.10 ± 0.36	Dry salting	-	-	46.2 ± 0.12	-	-	-	42.62 ± 0.55	-	(4–20 °C)
		Brine 7%	51.80 ± 0.39	52.01 ± 1.18	52.67 ± 0.60	53.55 ± 0.60	50.29 ± 0.18	50.31 ± 0.60	50.94 ± 0.10	52.53 ± 0.12	(7–10%) (7–13%)
		Brine 10%	50.15 ± 0.07	50.85 ± 1.20	49.95 ± 0.13	50.66 ± 0.28	49.32 ± 0.30	50.20 ± 1.06	49.26 ± 0.68	48.95 ± 0.1	(10–13%)
		Brine 13%	48.89 ± 0.75	49.10 ± 0.64	48.41 ± 0.18	48.02 ± 0.38	48.56 ± 0.55	47.93 ± 0.10	47.98 ± 0.84	45.53 ± 0.17	(4–20 °C)
Ash (%)	2.64 ± 0.11	Dry salting	-	-	4.13±0.11	-	-	-	4.15±0.22	-	NS
		Brine 7%	4.25 ± 0.01	4.44 ± 0.14	4.75 ± 0.06	4.87 ± 0.03	4.37 ± 0.02	4.47 ± 0.14	4.79 ± 0.02	5.09 ± 0.10	(7–10%) (7–13%)
		Brine 10%	5.12 ± 0.03	5.65 ± 0.07	5.91 ±0.06	6.03 ± 0.10	5.03 ± 0.03	5.62 ± 0.05	6.05 ± 0.03	6.34 ± 0.12	(10–13%)
		Brine 13%	6.01 ± 0.02	6.18 ± 0.02	6.89 ± 0.11	7.06 ± 0.10	5.80 ± 0.04	6.19 ± 0.03	6.58 ± 0.16	6.99 ± 0.03	(3–6 h) (3–24 h) (3–48 h) (6–24 h) (6–48 h) (24–48 h)
Salt (%)	0.10 ± 0.02	Dry salting	-	-	1.51 ± 0.13	-	-	-	1.61 ± 0.22	-	NS
		Brine 7%	2.17 ± 0.01	2.39 ± 0.11	2.77 ± 0.03	2.98 ± 0.04	2.15 ± 0.02	2.16 ± 0.10	2.79 ± 0.02	3.09 ± 0.07	(7–10%) (7–13%)
		Brine 10%	2.92 ± 0.15	3.72 ± 0.09	3.98 ± 0.07	4.08 ± 0.07	2.72 ± 0.09	3.63 ± 0.18	4.05 ± 0.25	4.21 ± 0.14	(10–13%)
		Brine 13%	3.68 ± 0.20	4.19 ± 0.07	4.89 ± 0.07	4.97 ± 0.10	3.86 ± 0.02	3.95 ± 0.08	4.69 ± 0.06	4.82 ± 0.11	(3–6 h) (3–24 h) (3–48 h) (6–24 h) (6–48 h) (24–48 h)
Acidity as lactic acid (%)	0.50 ± 0.02	Dry salting	-	-	0.52 ± 0.02	-	-	-	0.62 ± 0.06		(4–20 °C)
		Brine 7%	0.45 ± 0.03	0.40 ± 0.08	0.37 ± 0.05	0.32 ± 0.02	0.55 ± 0.06	0.48 ± 0.06	0.43 ± 0.05	0.35 ± 0.03	(4–20 °C)
		Brine 10%	0.47 ± 0.02	0.42 ± 0.05	0.42 ± 0.03	0.35 ± 0.03	0.53 ± 0.08	0.52 ± 0.06	0.47 ± 0.03	0.40 ± 0.03	(3–6 h) (3–24 h)
		Brine 13%	0.50 ± 0.06	0.40 ± 0.06	0.37 ± 0.05	0.37 ± 0.03	0.55 ± 0.06	0.45 ± 0.06	0.43 ± 0.05	0.42 ± 0.05	(3–48 h) (6–48 h)

**Table 2:** Inorganic elements (mg/100g) of Halloumi cheese salted by different methods at 4 and 20 °C for different times (means of three trials ± SD).

Inorganic Elements	No Salt	Salting Method	Temperature of Cheese Salting (°C)								Pairs of Means That Significantly Differed at (*p* ≤ 0.05)
			4				20				
			Salting time (h)								
			3	6	24	48	3	6	24	48	
Na	25 ± 2	Dry salting	-	-	558 ± 133	-	-	-	568 ± 84	-	(4–20 °C)
		Brine 7%	741 ± 200	579 ± 48	1097 ± 154	1042 ± 80	694 ± 141	806 ± 241	1023 ± 204	1107 ± 144	20 °C→(3–6 h) (3–24 h) (3–48 h) (6–48 h) 4 °C→(3–24 h) (3–48 h) (6–24 h)
		Brine 10%	1141 ± 113	1436 ± 68	1430 ± 95	1539 ± 190	1042 ± 101	1360 ± 193	1489 ± 127	1625 ± 186	
		Brine 13%	1347 ± 384	1477 ± 358	1742 ± 362	1641 ± 218	1233 ± 330	1447 ± 300	1527 ± 210	1647 ± 355	
K	64 ± 6	Dry salting	-	-	71 ± 6	-	-	-	71 ± 10	-	(4–20 °C)
		Brine 7%	30 ± 6	26 ± 17	19 ± 6	18 ± 10	34 ±17	29 ± 12	17 ± 7	19 ± 5	(3–6 h) (3–24 h) (3–48 h) (6–24 h)(6–48 h) (DS,−7%)(DS, −10%)(DS, −13%)
		Brine 10%	38 ± 6	30 ± 11	19 ±8	23 ± 6	39 ± 10	27 ± 7	14 ± 7	23 ± 14	
		Brine 13%	32 ± 13	27 ± 8	18 ±9	16 ± 9	26 ± 10	28 ± 16	17 ± 14	15 ± 11	
Ca	970 ± 90	Dry salting	-	-	1064 ± 80	-	-	-	1093 ± 124	-	(4–20 °C)
		Brine 7%	841 ± 111	992 ± 134	845 ± 114	780 ± 70	875 ± 24	924 ± 54	854 ± 67	882 ± 134	(6–24 h)(6–48 h) (DS, −7%)(DS, −10%)(DS, −13%)
		Brine 10%	926 ± 82	821 ± 101	898 ± 86	834 ± 76	1026 ± 115	1009 ± 137	913 ± 37	922 ± 113	
		Brine 13%	912 ± 39	847 ± 75	751 ± 82	857 ± 30	871 ± 109	1048 ± 281	869 ± 30	855 ± 177	
Mg	42 ± 3	Dry salting	-	-	47 ± 2	-	-	-	48 ± 4	-	NS
		Brine 7%	37 ± 5	36 ± 4	34 ± 6	33 ± 2	37 ± 3	37 ± 4	36 ± 5	34 ± 2	(3–6 h) (3–24 h) (3–48 h) (6–24 h)(6–48 h) (DS, −7%)(DS, −10%)(DS, −13%)
		Brine 10%	39 ± 1	36 ± 6	38 ± 3	35 ± 4	43 ± 4	40 ± 4	30 ± 12	38 ± 6	
		Brine 13%	36 ± 9	36 ± 7	32 ± 4	33 ± 5	38 ± 8	40 ± 8	34 ± 5	34 ± 7	
P	470 ± 30	Dry salting	-	-	519 ± 41	-	-	-	519 ± 29	-	(4–20 °C)
		Brine 7%	442 ± 21	403 ± 13	391 ± 21	402 ± 22	488 ± 40	442 ± 26	436 ± 8	445 ± 22	3–6 h) (3–24 h) (3–48 h) 4°C→(7–13%) (DS, −7%)(DS, −10%)(DS, −13%)
		Brine 10%	435 ± 39	420 ± 30	436 ± 39	400 ± 7	469 ± 30	448 ± 26	426 ± 39	419 ± 3	
		Brine 13%	447 ± 24	422 ± 17	423 ± 11	457 ± 18	458 ± 10	440 ± 36	437 ± 16	457 ± 14	

DS: Dry salting.

**Table 3:** Textural characteristics of Halloumi cheese salted by different methods at 4 and 20 °C for different times (means of three trials ± SD).

Textural characteristics	No Salt	Salting Method	Temperature of Cheese Salting (°C)								Pairs of Means That Significantly Differed at (*p* ≤ 0.05)
			4				20				
			Salting Time (h)								
			3	6	24	48	3		24	48	
Hardness (Ν)	7.88 ± 0.39	Dry salting	-	-	10.52 ± 0.07	-	-	-	14.01 ± 1.38	-	(4–20°C)
		Brine 7%	6.85 ± 0.66	7.51 0.72	8.67 ± 0.09	9.11 ± 0.51	7.51 ± 1.04	8.25 ± 0.61	8.51 ± 1.22	9.39 ± 1.31	(7–10%) (7–13%) (10– 13%) (3–48 h)
		Brine 10%	9.90 ± 0.53	11.04 ± 1.19	11.48 ± 0.06	11.92 ± 0.94	9.93 ± 0.20	10.08 ± 0.64	11.94 ± 0.86	12.43 ± 1.88	
		Brine 13%	10.16 ± 0.35	10.63 ± 0.02	12.82 ± 1.18	15.46 ± 2.37	11.33 ± 0.83	11.24 ± 1.06	16.02 ± 1.38	19.54 ± 2.21	
Fracturability (N)	4.80 ± 0.55	Dry salting	-	-	8.07 ± 0.53	-	-	-	10.27 ± 0.53	-	(4–20°C)
		Brine 7%	4.65 ± 0.32	5.50 ± 0.322	6.04 ± 0.24	6.68 ± 0.64	6.63 ± 0.75	6.16 ± 0.46	7.29 ± 0.21	8.43 ± 1.21	(7–10%) (7–13%) (10–13%) (3–48 h) (6–48 h)
		Brine 10%	7.15 ± 0.33	7.83 ± 0.4	9.01 ± 0.17	10.30 ± 0.24	7.76 ± 0.16	8.45 ± 0.38	10.51 ± 0.73	12.16 ±0.44	
		Brine 13%	8.41 ± 0.09	9.65 ± 0.15	11.10 ±0.41	13.91 ± 0.47	9.07 ± 0.14	10.66 ±0.31	13.88 ± 1.11	18.72 ±1.65	
Adhesiveness (Ν.mm)	6.83 ± 3.07	Dry salting	-	-	11.30 ± 2.08	-	-	-	7.00 ± 0.66	-	(4–20°C)
		Brine 7%	10.58 ± 3.41	4.59 ± 1.03	9.63 ±2.23	11.93 ± 3.89	9.52 ± 0.47	5.47 ±0.33	7.67 ± 2.17	9.42 ± 3.27	(7–13%)
		Brine 10%	11.95 ± 3.14	8.09 ± 1.54	10.43 ± 3.07	12.04 ±1.93	11.73 ±1.3	11.10 ± 2.74	7.67 ± 1.34	9.59 ± 4.68	
		Brine 13%	13.15 ± 4.16	10.76 ± 2.19	12.76 ± 3.6	17.24 ± 1.56	12.25 ± 1.73	11.74 ± 5.73	10.38 ± 3.1	12.11 ± 5.65	
Elasticity (mm)	0.95 ± 0.01	Dry salting	-	-	0.92 ± 0.02	-	-	-	0.91 ± 0.03	-	NS
		Brine 7%	0.93 ± 0.86	0.93 ± 0.01	0.94 ± 0.9	0.95 ± 0.01	0.93 ±0.86	0.94 ± 0.02	0.94 ± 0.01	0.95 ± 0.01	NS
		Brine 10%	0.93 ± 0.02	0.94 ± 0.02	0.94 ± 0.01	0.94 ± 0.01	0.93 ± 0.01	0.94 ± 0.86	0.94 ± 0.02	0.94 ± 0.02	NS
		Brine 13%	0.94 ± 0.02	0.94 ± 0.01	0.93 ± 0.02	0.93 ±0.02	0.92 ± 0.01	0.92 ± 0.01	0.93 ± 0.02	0.92 ± 0.01	NS
Cohesiveness	0.47 ± 0.01	Dry salting	-	-	0.48 ± 0.01	-	-	-	0.48 ± 0.01	-	NS
		Brine 7%	0.52 ± 0.01	0.48 ± 0.01	0.49 ± 0.01	0.50 ± 0.01	0.48 ± 0.01	0.48 ± 0.01	0.49 ± 0.01	0.48 ± 0.01	NS
		Brine 10%	0.51 ± 0.01	0.49 ± 0.01	0.49 ± 0.01	0.49 ± 0.01	0.49 0.01	0.49 ± 0.01	0.48 ± 0.01	0.48 ± 0.01	NS
		Brine 13%	0.49 ± 0.01	0.50 ± 0.01	0.48 ± 0.01	0.49 ± 0.01	0.48 ± 0.01	0.49 ± 0.01	0.48 ± 0.01	0.47 ± 0.01	NS
